# The Removal of Hydrophobic Matter from Thermosensitive Poly[*oligo*(ethylene glycol) Monomethyl Ether Acrylate] Gel Adsorbent in Alcohol–Water Mixtures

**DOI:** 10.3390/gels8040200

**Published:** 2022-03-23

**Authors:** Syed Ragib Safi, Toshiki Kaneko, Katsuhiro Nakahara, Takehiko Gotoh, Takashi Iizawa

**Affiliations:** Chemical Engineering Program, Graduate School of Advanced Science and Engineering, Hiroshima University, 1-4-1 Kagamiyama, Higashi Hiroshima, Hiroshima 739-8527, Japan; safi144@gmail.com (S.R.S.); tiizawa@hiroshima-u.ac.jp (T.I.)

**Keywords:** thermosensitive, LCST, UCST, hydrophobic, alcohols

## Abstract

A thermosensitive gel that exhibits lower critical solution temperature (LCST) becomes hydrophilic at low temperatures and hydrophobic at high temperatures in water. A system for absorbing hydrophobic organic matters that exploits this property has been reported. While washing the gel at a low temperature with a good solvent is a possible method for removing the adsorbed matter, a process that then shrinks the gel is also required. Herein, we focused on poly[*oligo*(ethylene glycol) mono(m)ethyl ether acrylate] (POEGA) gels as thermosensitive gels suitable for use in this system. POEGAs are known to contain poly(ethylene glycol) (PEG) units in their side chains and exhibit upper critical solution temperature (UCST) behavior in aliphatic alcohols. By exploiting this property, we developed a method for removing hydrophobic matters that accumulate in these gels; we also evaluated the LCST and UCST behavior of POEGA gels in alcohol–water mixtures, and measured the LCSTs of these gels in water and their UCSTs in some alcohols.

## 1. Introduction

Since the 1950s, thermosensitive polymers have attracted significant research attention in fields ranging from drug delivery to engineering [1], which is attributable to their unique thermal behavior in solution. For example, poly(*N*-isopropylacrylamide) (PNIPA) undergoes solubility-decreasing structural reordering upon heating in aqueous solution [1,2]; the temperature at which this reordering occurs is known as the “lower critical solution temperature” (LCST) or “cloud point”. Recently, poly[*oligo*(ethylene glycol) monomethyl ether acrylate] (POEGA) and poly[*oligo*(ethylene glycol) monomethyl ether methacrylate] (POEGMA) have gained attention as thermosensitive polymers that may potentially replace the widely used PNIPA [2,3,4,5], which is ascribable to their biocompatible acrylate or methacrylate main chains and pendant *oligo*(ethylene glycol) side chains. These linear polymers and gels have additional advantages: their LCSTs are easily tuned, they display upper critical solution temperature (UCST) type transition behavior in aliphatic alcohols, and they are highly thermosensitive in very concentrated aqueous salt solutions. These polymers are easily prepared through the radical polymerization of the corresponding monomers, and their LCSTs can be controlled by adjusting the lengths of their *oligo*(ethylene glycol) chains without the addition of any co-monomer [2,4]. Furthermore, the LCSTs can be precisely adjusted by randomly copolymerizing either *oligo*(ethylene glycol) monomethyl ether acrylate (OEGA) monomers [6] or *oligo*(ethylene glycol) methyl monoether methacrylate (OEGMA) monomers [7] with different chain lengths.

Recently, Roth et al. reported that POEGMA_300_ [8] prepared by the RAFT polymerization of OEGMA_300_ (*M_n_* = ~300 g mol^−1^) and doubly thermosensitive diblock copolymers [9,10] consisting of POEGMA_300_ and PNIPA showed UCST-type transition behavior in aliphatic alcohols, while they exhibit LCST-type transition behavior in water. In addition, normal POEGA and POEGMA exhibited UCST-type transition behavior in an aliphatic alcohol, and their UCST as well as LCST could control the length of the pendant *oligo*(ethylene glycol) chain.

**Scheme 1:**
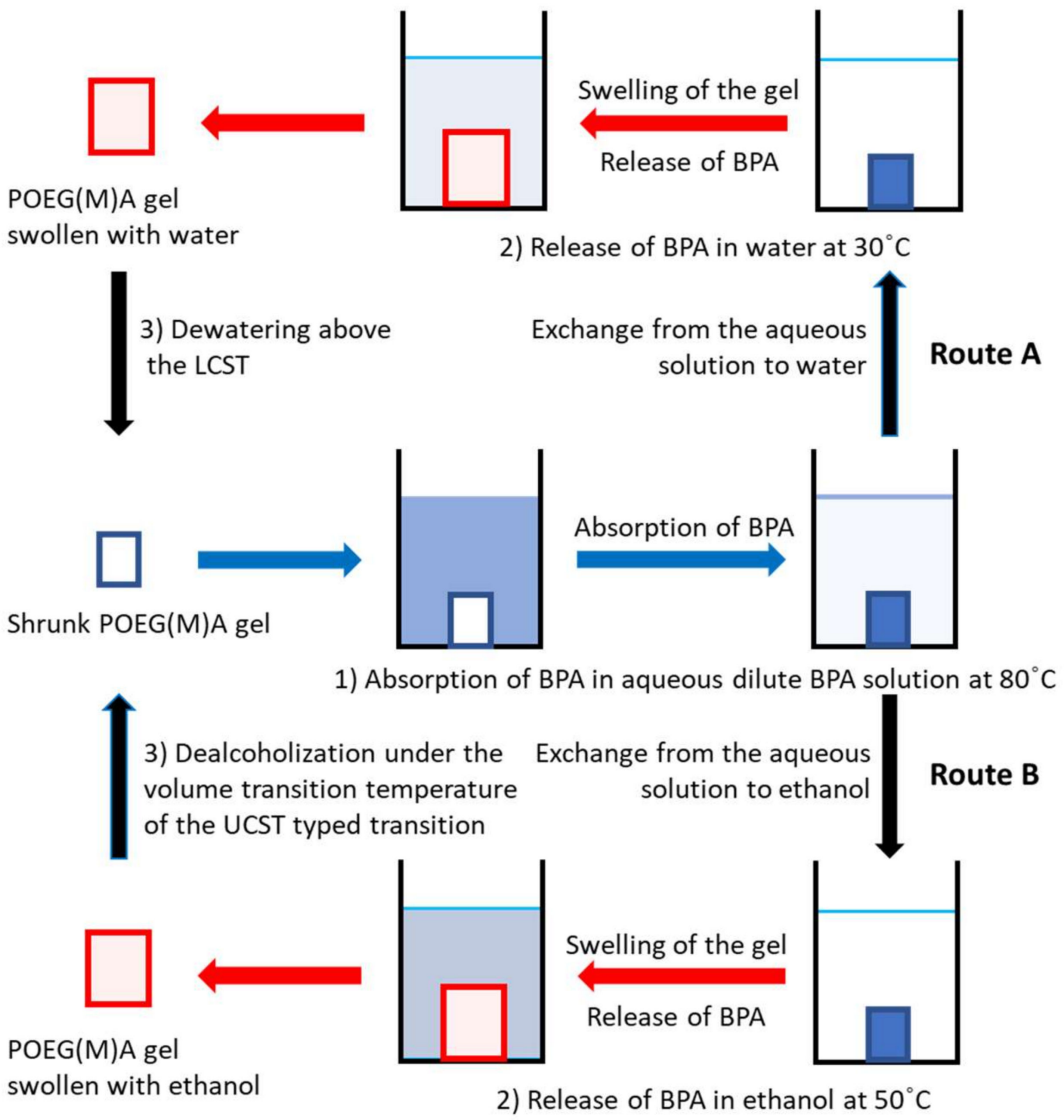
Novel process for removing hydrophobic impurities, such as BPA, from a dilute aqueous solution using porous POEG(M)A gels by regenerating shrunken gels based on their LCST- (**Route A**) and UCST-type (**Route B**) transition behavior.

We previously reported [11,12] the syntheses of porous POEGA and POEGMA gels by the thermal phase-separation polymerization of the corresponding OEGA and OEGMA monomers, and evaluated their phase-transition behavior on the basis of the ratios of the equilibrium external radii of the fully swollen to shrunken gels as functions of temperature in pure water and aqueous NaCl or CaCl_2_ solutions, despite the turbidimetrically determined LCSTs of the corresponding linear polymers being known [2,4,5]. The resulting porous gels also exhibited unique properties, including rapid swelling–deswelling behavior and high thermosensitivity in highly concentrated aqueous NaCl or CaCl_2_ solution, while the LCSTs determined from the equilibrium swelling ratio decreased with increasing NaCl or CaCl_2_ concentration. In addition, we examined a process for removing oil from an oil–water emulsion based on the LCST behavior of the obtained porous POEG(M)A gels. This process was shown in Scheme 1. (1) The shrunken gel adsorbs oil in the oil–water emulsion. There are two routes to reproduce the gel shrunken and adsorbed impurities to the original shrunken gel, each consisting of two steps: (2) washing out the impurities by swelling the gel, and (3) delivering gels swollen with solvent based on the LCST-type (Route A) and UCST-type (Route B) transition behavior. However, Route A did not proceed smoothly, although the hydrophobic shrunken gel adsorbs oil because the POEG(M)A gel acts as a polymeric interfacial activator below the LCST, and the impurity is very insoluble in water. A workaround to this problem involves using the UCST-type transition (route B) because POEG(M)A gel did not work as a polymeric interfacial activator and the solubility of the impurity is high in ethanol.

Bisphenol-A (BPA) is an endocrine disruptive compound (EDC) that may mimic or interfere with the endocrine or hormonal system [13]. Some effects of BPA on health are sexual dysfunction and fertility; metabolic disease, such as obesity; cancer (breast cancer, neuroblastoma); neurological effects (disruption of the dopaminergic system); cardiovascular diseases; thyroid disorders and asthma [14,15]. Various studies have been conducted previously to separate BPA from water. Some of the significant techniques include coagulation, flocculation, precipitation, and other separation methods to remove bisphenol A from water. Except adsorption, most of the other techniques have drawbacks such as lack of efficiency, increased operational cost, generation of toxic byproducts, and inability to reuse or recycle materials. Hence, adsorption technology is the most commonly used technique for BPA removal [16] as well as benzidine [17]. There are a variety of adsorbents to remove BPA, such as activated carbon [18,19], clays [20,21,22,23], zeolites [24,25], chitosan [26,27], agricultural wastes [28,29], and biochar [30,31]. Hydrogels could also adsorb BPA from water [32,33]. However, these adsorbents were hard to regenerate and require much energy to desorb BPA from the adsorbents. Therefore, thermosensitive polymers and gels have attracted attention as a reversible adsorbent to remove hydrophobic contaminants from water. Nakano et al. reported temperature-sensitive heteropolymer gels synthesized with sodium styrene sulfate (SSS) and vinylbenzyl trimethylammonium chloride (VBTA) [34]. The SSS-VBTA gel shows the thermo-reversible changes in its volume and properties at around room temperature. The SSS-VBTA gel could concentrate trace amounts of Bisphenol-A present in very diluted aqueous solutions at around room temperature and release the concentrated Bisphenol-A above room temperature. However, it is sometimes difficult to remove the adsorbed matter from the swollen gel at a lower temperature than LCST [12] because some of the impurities were absorbed or distributed in the hydrogel and have higher affinity with polymer than water. Therefore, the gel needs to be washed in a good solvent to remove impurities. Herein, we examined the transition behavior of porous POEG(M)A gels in some aliphatic alcohols and alcohol–water mixtures. We report the removal of bisphenol A (BPA), as a hydrophobic impurity, from a dilute aqueous solution using POEG(M)A porous gels and by regenerating shrunken gels based on their LCST- and UCST-type transition behavior (Scheme 1) in water and in some alcohols, respectively.

## 2. Results and Discussion

### 2.1. Transition Behavior of POEG(M)A Gels in Water and Alcohol

**Figure 1:**
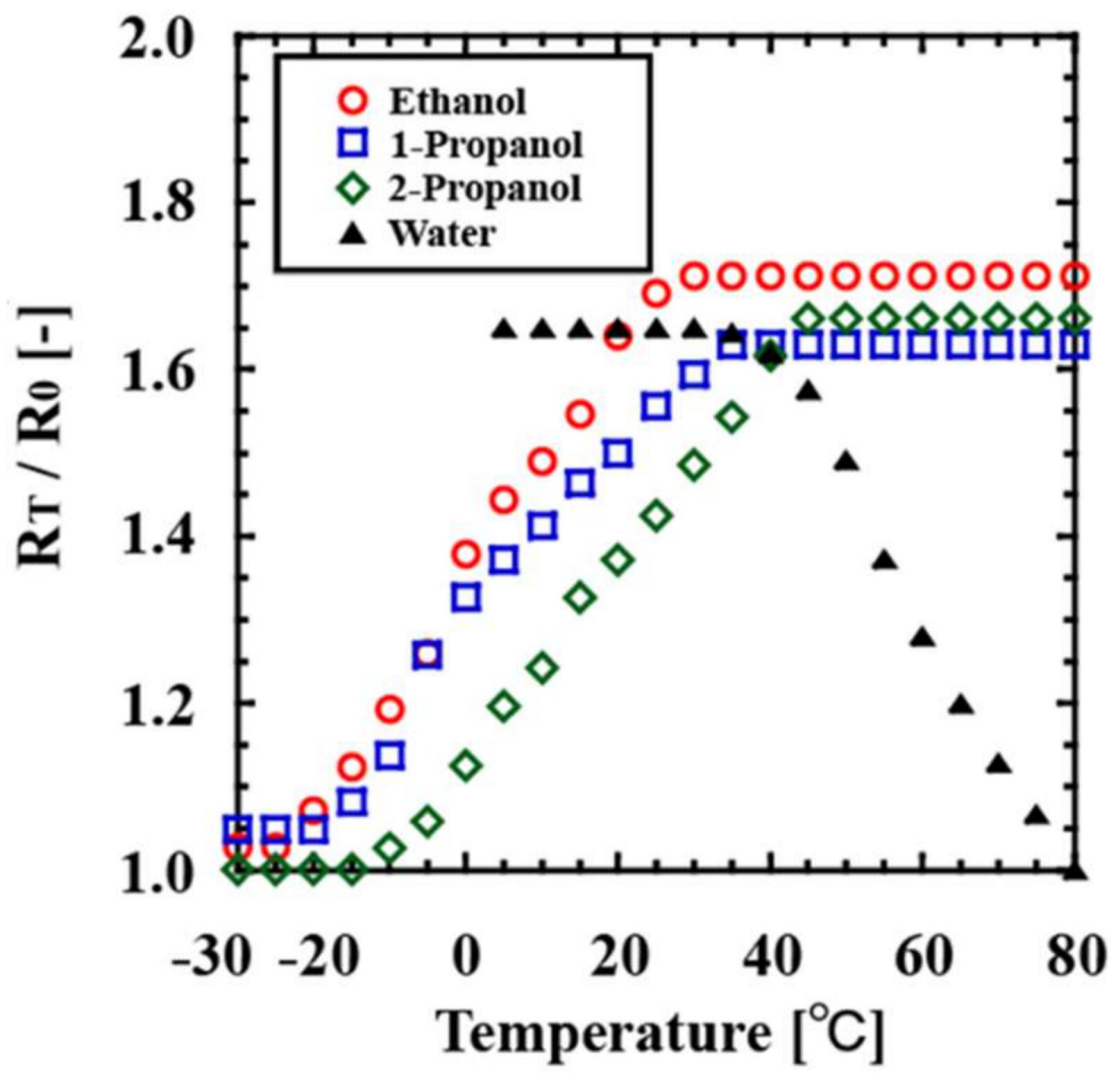
Transition behavior of PTEGA in pure water and various alcohols.

**Figure 2:**
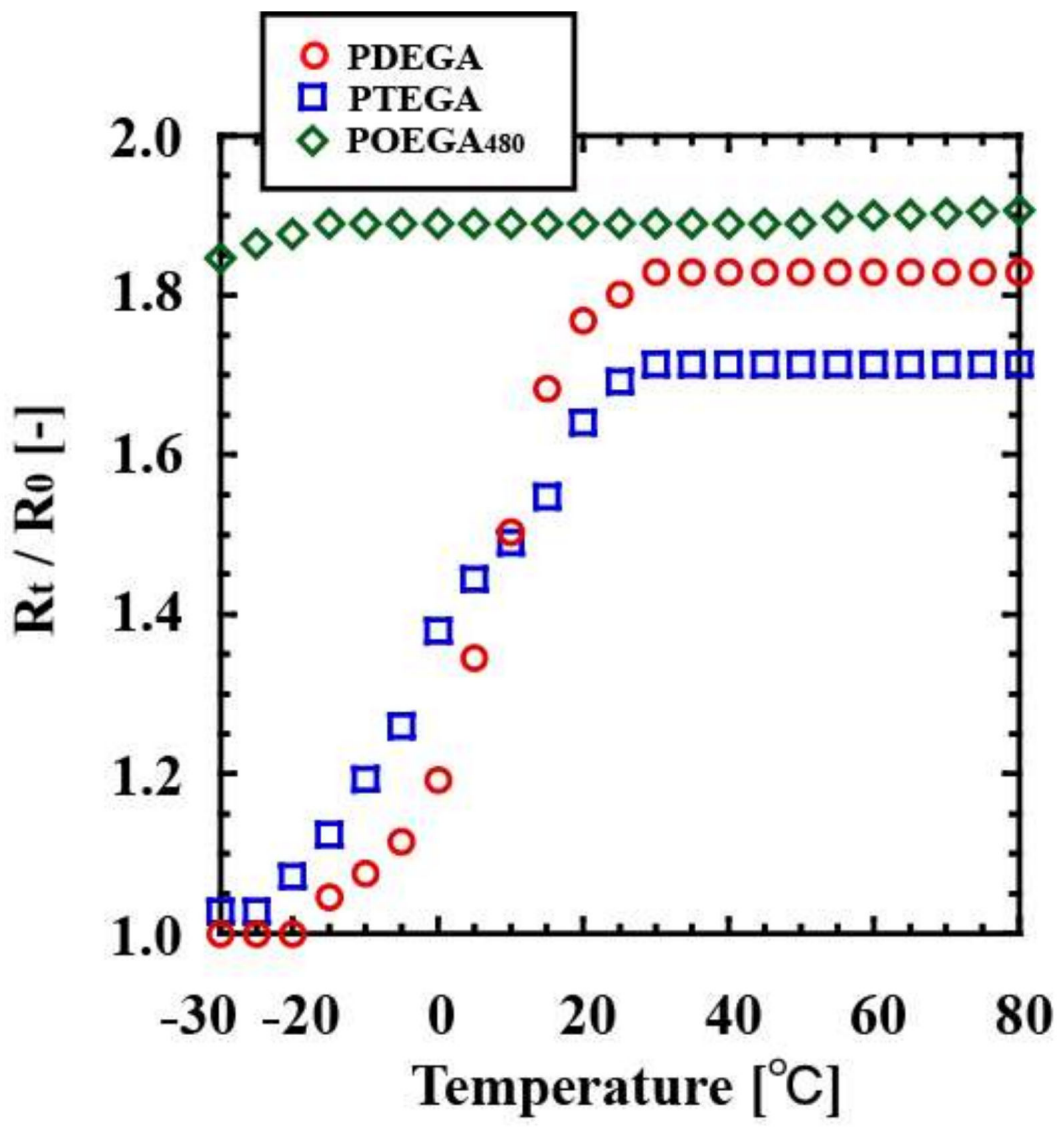
Transition behavior of some gels in ethanol. ○; Poly[di(ethyleneglycol) monoethylether acrylate (PDEGA), □; Poly[tri(ethyleneglycol) monomethyl etheracrylate] (PTEGA), ◇; Poly[oligo(ethylene glycol) monomethyl etheracrylate] (PTEGA480).

POEG(M)A sponge-like porous gels were prepared by the radical phase separation polymerization of the corresponding monomers according to a previously reported method (Table 1) [11,12]. PDEGA, PDEGMA, and PTEGMA porous gels were obtained using suitable ethanol–water mixtures as polymerization solvents because their monomers are poorly water soluble. POEGA_300_ was also obtained using an aqueous 8 wt% NaCl solution to prevent swelling at 80 °C. The transition behaviors of the POEG(M)A porous gels were investigated in water and various alcohols, with the temperature-dependent swelling behavior of the PTEGA gel shown in Figure 1. The UCST-type behavior of the porous gel was determined from the equilibrium swelling ratio (R_T_/R_0_) at each temperature [12], where R_0_ was the external radius R_0_ of the gel shrunken in water at 80 °C and R_T_ was the swollen equilibrium radius at each temperature T (°C). The PTEGA gel adsorbs water at low temperatures and swells in pure water; it also desorbs water and shrinks at high temperatures. These swelling and shrinking characteristics express the change of hydrophobicity of the hydrogel according to the temperature as well as the contact angle. (Figure S2a,b). During the process, the porous gel kept its sponge like morphology to swell and shrink fast as we reported before [11,12]. These features are indicative of typical LCST-type transition behavior [12]. On the other hand, the gel shrank and did not adsorb ethanol, 1-propanol, or 2-propanol at low temperatures. The gel began to swell at a certain temperature (the volume transition temperature) with increasing alcohol temperature; it adsorbed alcohol and became effectively swollen at higher temperatures, indicative of UCST-type transition behavior [35]. Similar behavior was observed for the POEGMA_300_ polymer and its diblock copolymers [8,9,10]. The temperature-dependent swelling behavior of the PDEGA, PTEGA, and POEGA_480_ gels in ethanol is shown in Figure 2. The POEGA_300_ gel is known to exhibit UCST-type transition behavior in water [8]. The PDEGA and PTEGA gels exhibited almost identical UCSTs of approximately 22 °C, despite swell-start temperatures of −25 °C and −20 °C, respectively, while the UCST of the POEGA_480_ gel (bearing long pendant PEG chains) in ethanol is below −20 °C, and could not be determined with the equipment used in this study. Therefore, the UCST of the POEGA gel in ethanol decreases with increasing PEG side-chain length; hence, the UCST shifts to lower temperature with increasing PEG chain length in order to increase hydrophilicity. These results suggest that POEGA gels with pendant PEG chains exhibit LCST-type transition behavior in water and UCST-type transition behavior in aliphatic alcohols.

**Figure 3:**
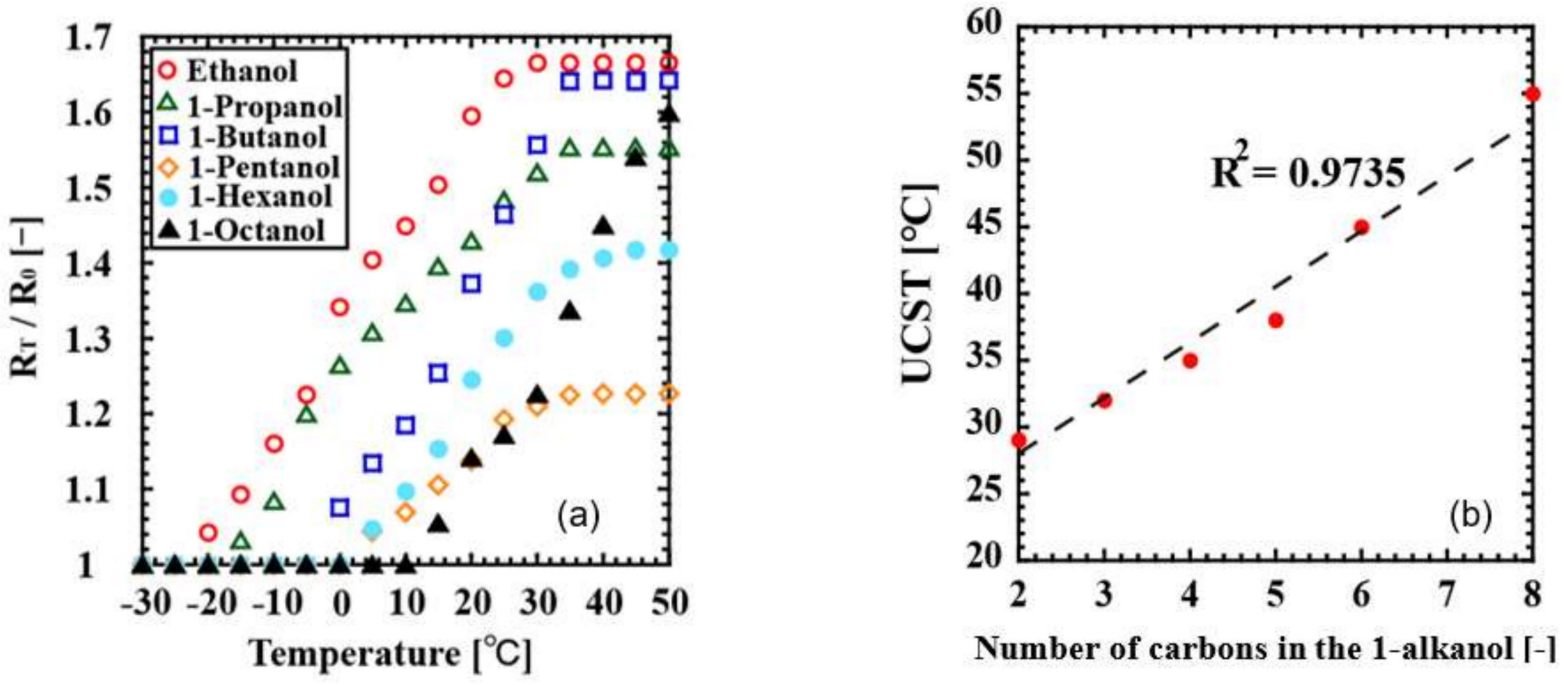
(**a**) UCST-type transition behavior of PTEGA gels in 1-alkanols. (**b**) UCST of the PTEGA gel as a function of the length of the 1-alkanol.

Figure 3a shows the transition behavior of the PTEGA gel in six 1-alkanols. The transition temperature was observed to shift to higher temperatures with increasing alkyl chain of the alcohol because hydrophobicity increases as the length of the alcohol alkyl chain increases. Figure 3b shows the relationship between the number of carbons in the alkyl chain of the PTEGA gel and the UCST. POEGMA_300_ reportedly shows a linear correlation between the UCST and the length of the alcohol chain [8]. The volume transition temperature and the UCST of the PTEGA gel linearly correlate with the size of the alcohol. In addition, the PTEGA gel did not swell in ethanol, 1-butanol, and 1-octanol at their corresponding start-swell temperatures of −25, −10, and 10 °C, respectively. Therefore, these solvents can be separated from the swollen gels when cooled to the volume transition temperature.

**Figure 4:**
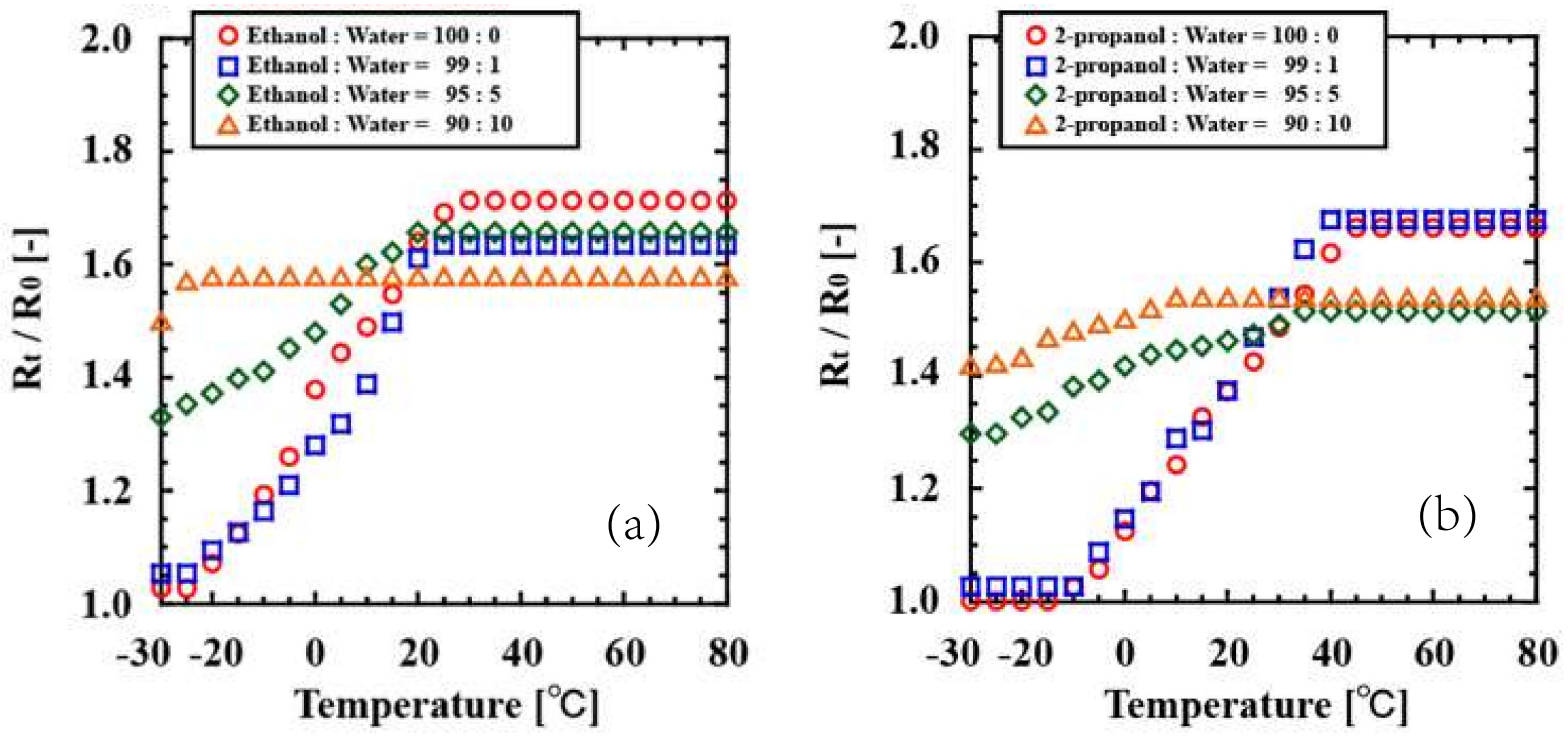
UCST behavior of the PTEGA gel in (**a**) hydrous ethanol and (**b**) hydrous 2-propanol.

**Figure 5:**
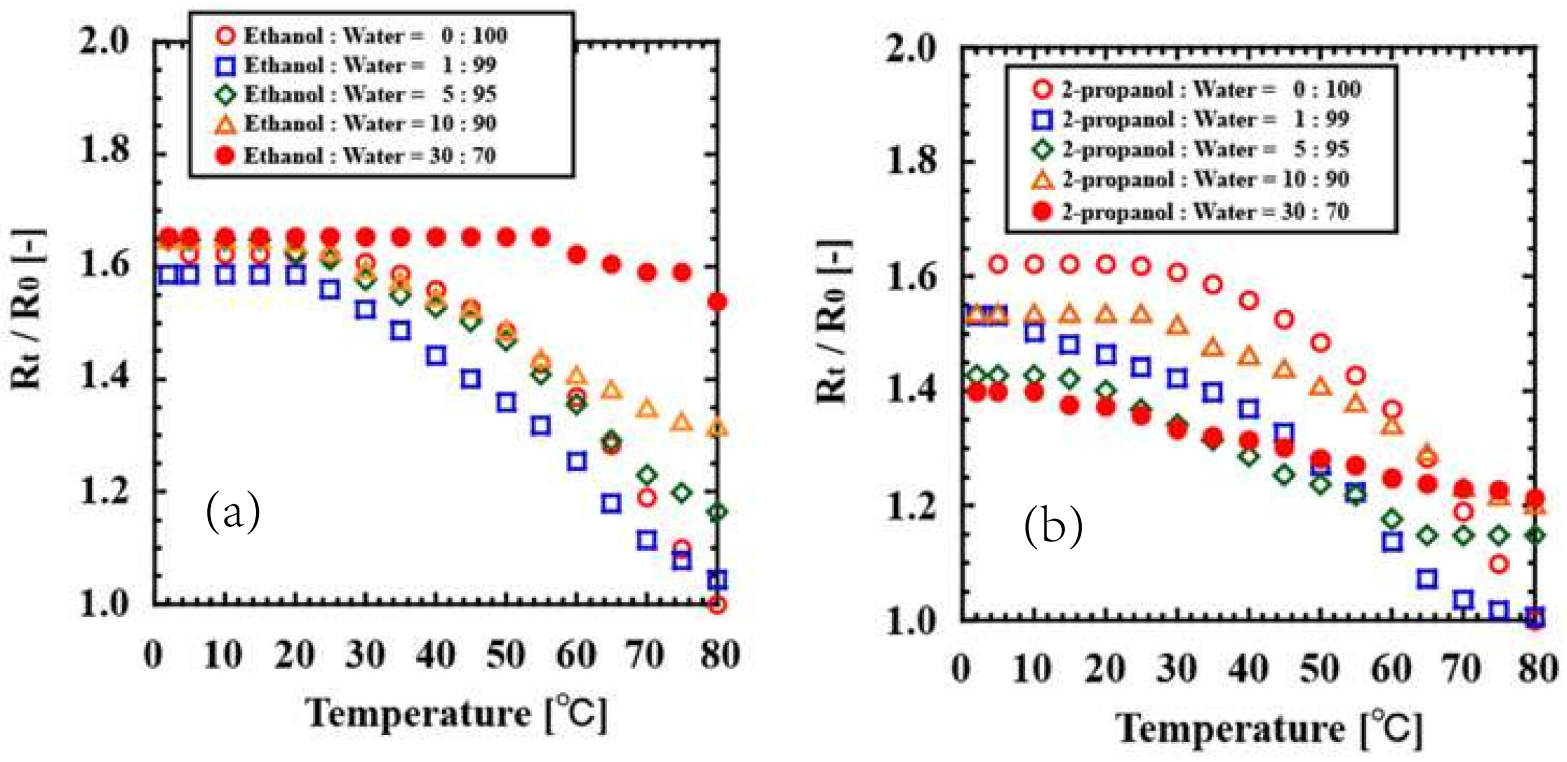
LCST behavior of the PTEGA gel in (**a**) aqueous ethanol and (**b**) aqueous 2-propanol.

Understanding how impurities in the solvent affect the abovementioned thermosensitivities is very important for gel applications. Figure 4a,b show how the addition of water to the alcohol system affects the UCST and thermal properties of the gel. UCST-type thermosensitivity was observed to hardly change when a small amount of water (≤1 wt%) was added to ethanol or 2-propanol; however, the UCST and thermosensitivity were observed to decrease at ≥5 wt%, and only small differences were observed between ethanol and 2-propanol. In contrast, how the addition of alcohol to water affects the LCST and thermal properties of the gel are shown in Figure 5a,b; LCST-type thermosensitivity was observed to decrease with increasing ethanol or 2-propanol concentration in water, with the addition of ethanol appearing to affect thermosensitivity less than 2-propanol. These results reveal that the solvent must be highly pure to ensure that the gel exhibits sharp transition behavior in the solvent.

### 2.2. Adsorption and Desorption of BPA by the POEGA and POEGMA Gels

Scheme 1 shows the novel process for removing hydrophobic impurities from dilute aqueous solutions using POEG(M)A porous gels, which involves regenerating shrunken gels by exploiting UCST-type transition behavior; these gels have high affinities for a variety of chemicals. POEG(M)A gels shrunken below the LCST are reportedly useful adsorbers of chemicals, such as petroleum oil [12], protein [36], and dye [37] by hydrophobic interaction as we expressed in our previous study [12]. The BPA is a hydrophobic organic material [38] and can be adsorbed by thermosensitive gel [39] including PEGDA [40] when the gel is hydrophobic. Therefore, BPA (40 ppm) was adsorbed from water by the POEG(M)A gels (i.e., PDEGMA, PTEGMA PDEGA, and PTEGA) at 80 °C over 48 h; these gels were hydrophobic and adsorbed BPA as their LCSTs are lower than this temperature. However, the gels were dipped in pure water at 30 °C for 48 h, during which time they became hydrophilic and were unable to adsorb BPA; consequently, the BPA was released from each gel. While the POEGMA gels adsorbed BPA better than the corresponding POEGA gels, the former desorbed less than the latter, with BPA not released, as expected, consistent with similar previous reports [12]. Hydrophobic impurities are removed with difficulty from thermosensitive gels when their transitions in water are relied on. On the other hand, the gels swelled well in 50 °C ethanol instead of pure water, with BPA removed perfectly from each gel. In addition, ethanol was easily removed from the swollen PDEGA and PTEGA gels when cooled to −20 °C and −25 °C, respectively. In order for this adsorption and desorption process to operate smoothly, the solvent needs to remain highly pure because the gel exhibits sharp transition behavior in pure solvents (Figure 4 and Figure 5). Hence, this method is useful for the removal and collection of trace amounts of hydrophobic chemicals in water, as well as for washing gels contaminated with hydrophobic chemicals by optimizing the combination of the POEG(M)A gel, alcohol, and chemicals.

**Figure 6:**
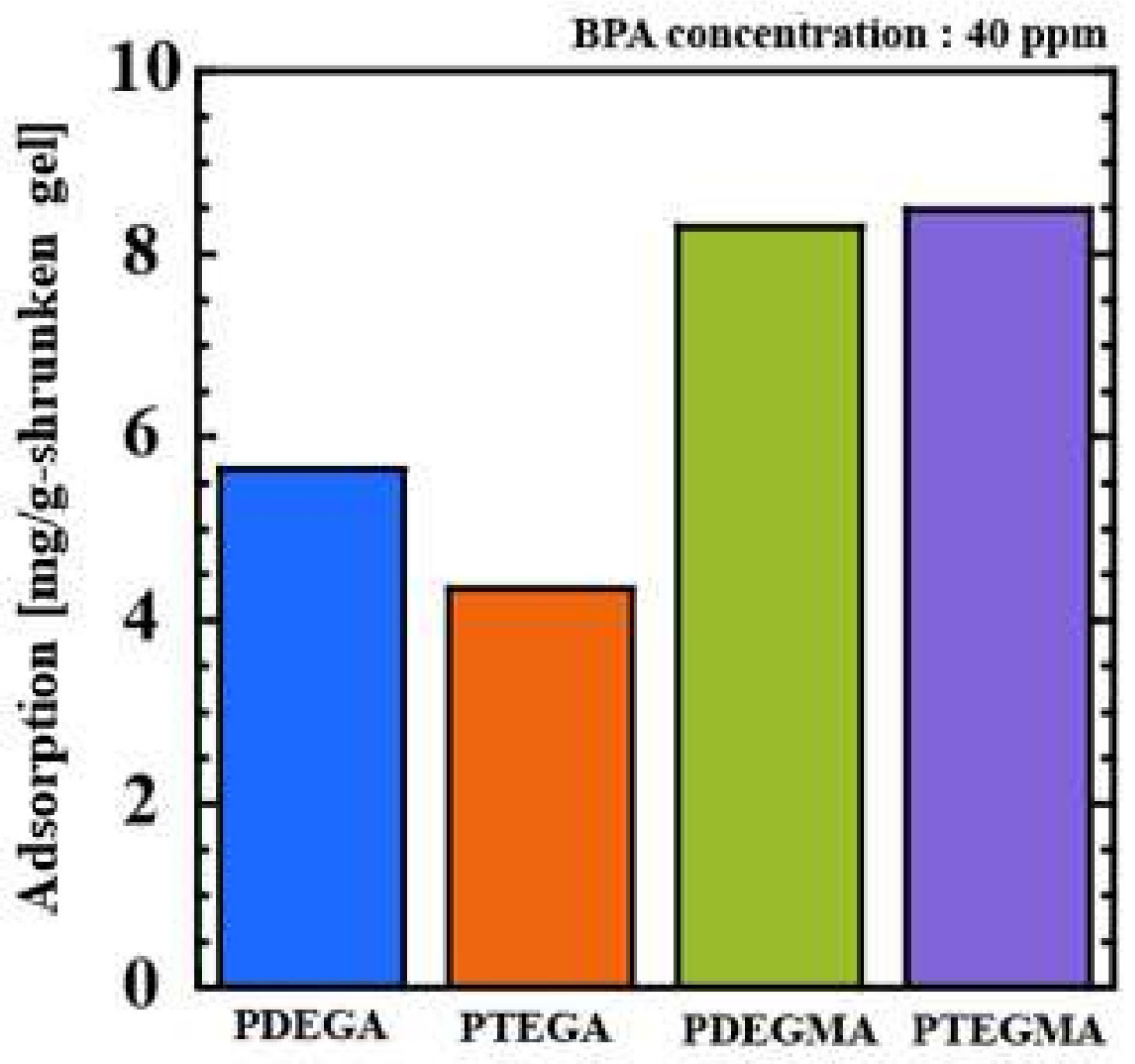
Adsorption of BPA by the POEGA and POEGMA gels.

**Figure 7:**
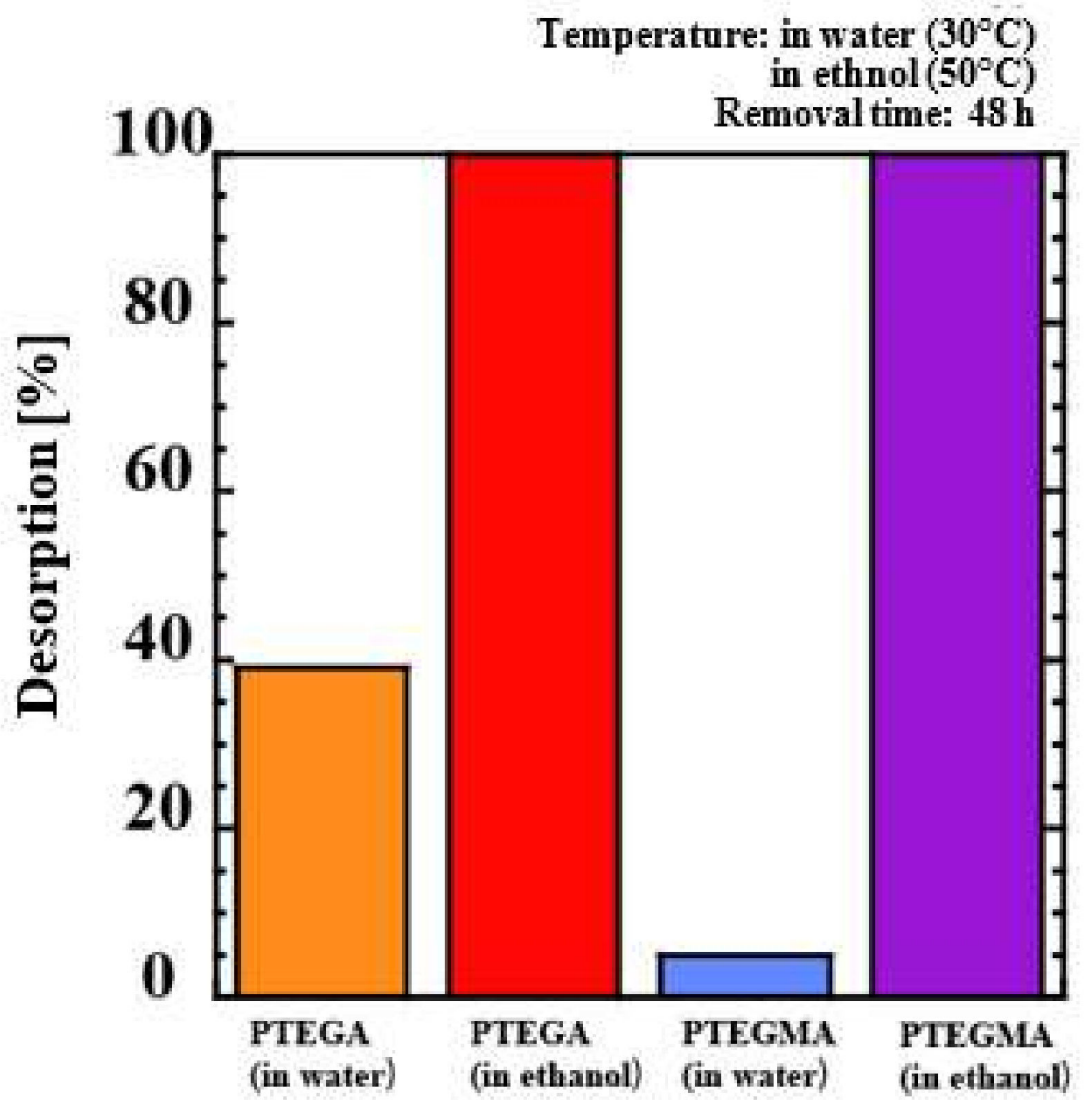
Desorption of BPA by the POEGA and POEGMA gels.

Figure 6 shows the adsorption of BPA by the POEGA and POEGMA gels. POEGMA gels adsorb better than POEGA gels because methacrylate gels are more hydrophobic than acrylate gels [11,12]. Figure 7 shows the desorption of BPA from the POEGA and POEGMA gels in water or in ethanol. PTEGMA gel desorbed the BPA less than PTEGA gel in water because PTEGMA gel was more hydrophobic than PTEGA gel and has higher affinity with BPA. On the other hand, both gels could desorb the BPA completely in ethanol. This shows the effectiveness of the usage of the alcohol to remove the hydrophobic impurities from the thermosensitive POEG(M)A gels for regeneration of them as the adsorbent.

## 3. Conclusions

The thermosensitive characteristics of a poly[tri(ethylene glycol) monomethyl ether acrylate] (PTEGA) gel in water and various pure alcohols were investigated. We revealed that this gel has an LCST of 80 °C in water, and UCSTs in pure aliphatic alcohols, such as ethanol, 1-propanol, and 2-propanol. In addition, the UCST was observed to increase with increasing alcohol chain length. The UCST of the POEGA gel in ethanol was observed to decrease with increasing PEG side-chain length, with the UCST of POEGA_480_ (bearing a long PEG chain) was ≤−30 °C and could not be measured. In addition, the thermosensitive characteristics were observed to hardly change when a small amount of water (~1 wt%) was added to ethanol, while the UCST was observed to decrease and the thermosensitive characteristics were observed to increase at ≥5 wt%. On the other hand, a small amount (a few wt%) of ethanol added to pure water had almost no effect on the observed LCST characteristics; however, the LCST was observed to increase and the thermosensitivity was observed to decrease at >10 wt%. Based on these results, we conclude that hydrophobic substances, such as BPA, adsorbed on the gel are removed perfectly by immersing them in ethanol or 2-propanol, and by washing the gel at below −20 °C to remove the ethanol or 2-propanol. The gel can be regenerated to adsorb more BPA by immersing it in 80 °C water.

## 4. Materials and Methods

### 4.1. Materials

Di(ethylene glycol) monomethyl ether acrylate (DEGA), di(ethylene glycol) monoethyl ether acrylate (eDEGA), tri(ethylene glycol) monomethyl ether acrylate (TEGA), *oligo*(ethylene glycol) monomethyl ether acrylate (OEGA_480_, *M_n_* = ~480 g mol^−1^), and di(ethylene glycol) diacrylate (DEGDA) were purchased from the Hitachi Chemical Co. Ltd., Tokyo, Japan. Potassium peroxodisulfate (K_2_S_2_O_8_) and anhydrous sodium sulfite (Na_2_SO_3_) were purchased from the Katayama Chemical Co. Ltd., Osaka, Japan, and Wako Pure Chemical Industries Ltd., Osaka, Japan, respectively. The monomers and initiators were used as received. Sodium chloride (NaCl) and anhydrous ethanol (≥99.5%) were purchased from Nacalai Tesque, Inc., Kyoto, Japan. All solvents used were purified by distillation. Prior to use, distilled water was sonicated for 30 min to release dissolved gases. Activated charcoal granules (AC) were purchased from the Kanto Chemical Co. Inc., Tokyo, Japan. The chemical structures of monomers and crosslinkers are provided in Supplementary Materials.

### 4.2. Preparing the Thermosensitive Porous Gels

A typical procedure for the synthesis of a porous gel is described. TEGA (5.0 g, 23 mmol), DEGDA (98 mg, 0.46 mmol), and potassium peroxodisulfate (31 mg, 0.115 mmol) were dissolved in water (12 mL) and added to a 50-mL cylindrical flask containing approximately 25 Teflon tubes (internal diameter: 4 mm; length: ~3 cm; these tubes served as in-situ polymerization scaffolds). A solution of anhydrous sodium sulfite (29 mg, 0.23 mmol) in water (3 mL) was added to the flask under flowing nitrogen. The reaction mixture was stirred for 10 min and left to stand for 12 h at 80 °C to complete the gel polymerization process. After gelation was complete, the polymer-filled tubes were retrieved from the bulk gel in the flask by tweezers. The filled tubes were sliced crosswise to produce cylindrical specimens of equal length and diameter. Uniformly shaped gel samples were obtained by expelling the gels from the tubes, after which unreacted substances were removed by immersion in a large amount of methanol for 24 h. Each gel was then immersed in a large amount of water to remove the methanol for 24 h, with the water replaced three times in 24 h. The resulting white sponge-like PTEGA pellets were placed in water and stored in their swollen conditions in a refrigerator at 5 °C. The gels prepared using this procedure are summarized in Table 1.

### 4.3. Examining the LCST and UCST Behavior of the Porous Gels

A stored porous gel was dipped in hot (80 °C) water, after which the external radius (R_0_) of the resulting shrunken gel was measured. The gel was then swelled in a large amount of pure alcohol or an alcohol–water mixture at 50 °C to replace the water in the gel with the solvent. The swollen gel was then reshrunk at −30 °C.

The shrunken porous gel was immersed in a different solvent at −30 °C, and the swollen equilibrium diameter (2R_T_) at each temperature *t* (°C) was measured in the −30 °C to 80 °C range using a low-temperature constant temperature bath (solvent: ethanol, water). The UCST-type behavior of the porous gel was determined from the equilibrium swelling ratio (R_T_/R_0_) at each temperature. The LCST-type behavior in aqueous alcohol was also determined from R_T_/R_0_ values in the 0–80 °C range. The external radius (R_0_) of the gel shrunken in an aqueous 5 wt% NaCl solution at 80 °C was adopted for the POEGA_480_ gel because the gel was swollen in pure water at 80 °C [12].

### 4.4. Measurement of the Contact Angle of the Gel

The contact angle of the gel was measured by the half-angle method of drawing a line from the triphase point to the apex of a droplet. A pure water of 1 µL was measured and dropped by a micro syringe on the gel film with 0.5 mm thickness. The gel film was put on the glass plate, which was placed in a closed stainless chamber equipped with a glass window. The chamber was placed in the water bath regulated at a desired temperature. A photograph of each droplet was taken to measure the height (*h*) and diameter (2*r*) of the droplet on the gel film. The contact angles were calculated from the height and diameter of the droplet as follows.

tan θ = *h*/*r*, θ = 2 arctan (*h*/*r*) (1)

## Figures and Tables

**Table 1:** Porous thermosensitive gels used in this study ^a^.

Gel	Crosslinker	Degree of Crosslinking (mol%)	Solvent (wt%)
PDEGA (*n* = 2)	DEGDA	1	15 wt% Aqueous ethanol solution
PTEGA (*n* = 3)	DEGDA	1	Water
POEGA480 (*n* = 8–9)	DEGDA	1	8 wt% Aqueous NaCl solution
PDEGMA (*n* = 2)	DEGDMA	2	80 wt% Aqueous ethanol solution
PTEGMA (*n* = 3)	DEGDMA	2	20 wt% Aqueous ethanol solution
POEGMA300 (*n* = 4–5)	DEGDMA	2	Water
PNIPA	MBAA	1	20 wt% Aqueous DMF solution

^a^ The monomer solution (25 wt%) was polymerized with the crosslinking monomer (2 mol%) in the indicated solvent using K_2_S_2_O_8_–K_2_SO_3_ as initiators under N_2_ at 80 °C for 12 h. The *n* means number of EG in monomer of each gel.

## Data Availability

The datasets analyzed during the current study are available from the corresponding author upon reasonable request.

## Supplementary Materials

The following are available online at https://www.mdpi.com/article/10.3390/gels8040200/s1, Figure S1: The chemical structures of (a) monomer and (b) crosslinker; Figure S2: (a) The effect of temperature on contact angle of PTEGA gel, (b) the effect of temperature on transition behavior of PTEGA gel in pure water; Figure S3: Measurement of contact angle of a droplet.
