# Supplementary material for: The Removal of Hydrophobic Matter from Thermosensitive Poly[oligo(ethylene glycol) Monomethyl Ether Acrylate] Gel Adsorbent in Alcohol–Water Mixtures
Source: Gels. 2022 Mar 23;8(4):200. doi: 10.3390/gels8040200 (PMC9029373; doi:10.3390/gels8040200)
Supplement: Supplementary file 1 [file gels-08-00200-s001.zip › gels-1606118-supplementary.pdf]

gels

Supplementary

The removal of hydrophobic matter from thermosensitive poly[*oligo*(ethylene glycol) monomethyl ether acrylate] gel adsorbentin alcohol–water mixtures

Takehiko Gotoh[1,*], Toshiki Kaneko[2], Katsuhiro Nakahara[2], and Takashi Iizawa[1]

1. Table of contents of the manuscript
2. The chemical structures of monomers and crosslinker
3. The effect of temperature on contact angle and transition behavior of PTEGA gel in pure water.

1. *Table of contents of the manuscript*

1. Introduction
2. Results and Discussion
2.1. Transition of behavior of POEG(M)A gels in water and alcohol
2.2. Adsorption and desorption of BPA POEGA and POEGMA gels
3. Conclusions
4. Materials and Methods
4.1. Materials
4.2. preparing the themosensitive porous gels
4.3. Examining the LCST and UCST behavior of porous gels
4.4. Measurement of the contact angle of the gel

2. *The chemical structures of monomers and crosslinker*

(a)

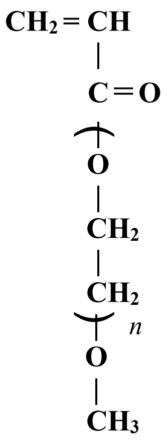

oligo(ethylene glycol) monomethyl ether acrylate, PDEGA (n = 2) PTEGA,(n = 3), POEGA480 (n = 8–9)

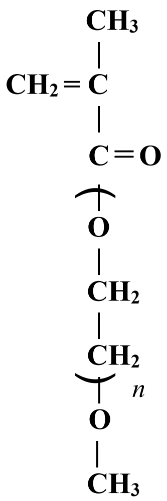

oligo(ethylene glycol) monomethyl ether methacrylate, PDEGMA (n = 2), PTEGMA (n = 3), POEGMA300 (n = 4–5)

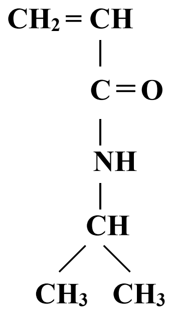

N-isopropylacrylamide

(b) **crosslinker**

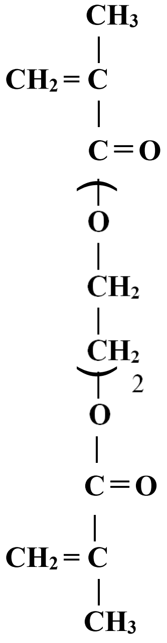

Diethyleneglycoldimethacrylate (DEGDMA)

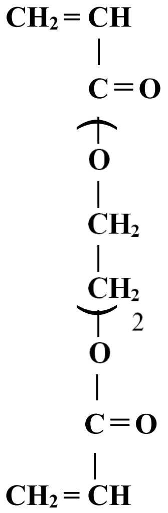

Diethyleneglycoldiacrylate (DEGDA)

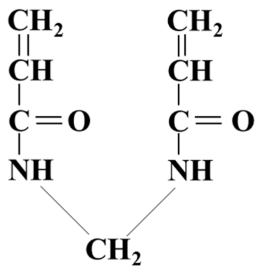

N,N '- methylenebisacrylamide (MBAA)

Figure S1. The chemical structures of (a) monomer and (b) crosslinker

3. The effect of temperature on contact angle and transition behavior of PTEGA gel in pure water.

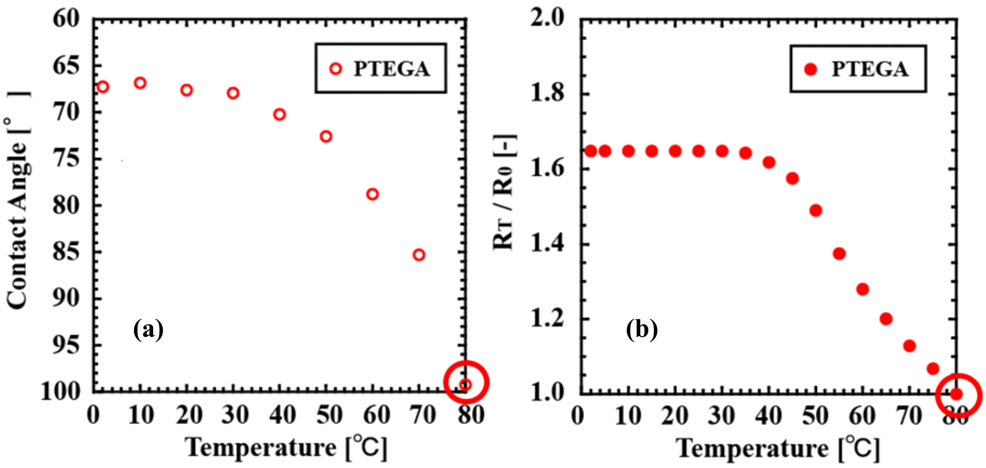

Figure S2. (a) The effect of temperature on contact angle of PTEGA gel, (b) the effect of temperature on transition behavior of PTEGA gel in pure water.

The contact angle of the gel was measured by the half-angle method of a drawing a line from the triphase point to the apex of a droplet. A pure water of 1 μl was measured and dropped by a micro syringe on the gel film with 0.5 mm thickness. The gel film was placed on the glass plate. The glass plate was placed in the chamber made of stainless steel equipped with a glass window. The chamber was placed in a water bath with temperature controller. A photograph of each droplet was taken to measure the height (h) and diameter (2r) of the droplet on the gel film. The contact angles were calculated from the height and diameter of the droplet as follows;

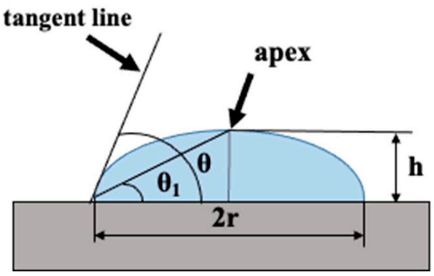

$$\tan \theta_1 = h/r, \quad \theta = 2 \arctan (h/r)$$

Figure S3. measurement of contact angle of a droplet.
